# Risk Modeling of Bat Rabies in the Caribbean Islands

**DOI:** 10.3390/tropicalmed5010035

**Published:** 2020-03-01

**Authors:** Clint N. Morgan, Ryan M. Wallace, Alexandra Vokaty, Janine F.R. Seetahal, Yoshinori J. Nakazawa

**Affiliations:** 1 Poxvirus & Rabies Branch, National Center for Emerging Zoonotic Infectious Diseases, Division of High Consequence Pathogens & Pathology, Centers for Disease Control and Prevention, 1600 Clifton Rd NE, Atlanta, GA 30333, USA; rmwallace@cdc.gov (R.M.W.); ynakazawa@cdc.gov (Y.J.N.); 2 Oak Ridge Institute for Science and Education, CDC Fellowship Program, 1299 Bethel Valley Rd, Oak Ridge, TN 37830, USA; 3 World Health Organization, India Country Office, RK Khanna Tennis Stadium, Africa Avenue, New Delhi 110029, India; vokatya@who.int; 4 Department of Preclinical Sciences, Faculty of Medical Sciences, The University of the West Indies, St. Augustine Campus, Building 36, Eric Williams Medical Sciences Complex, Champs Fleurs, Trinidad and Tobago; jseetahal@gmail.com

**Keywords:** lyssavirus, chiroptera, Latin America, risk mapping, surveillance, dispersal, zoonoses

## Abstract

Rabies surveillance and control measures vary significantly between Caribbean islands. The Centers for Disease Control and Prevention currently recommends certain groups of U.S. travelers to any Caribbean island receive pre-exposure rabies immunization. However, most islands self-declare as “rabies free”, and have never publicly released data to support rabies-free claims. We used the Analytic Hierarchy Process to create pairwise comparison values among five risk factors determined by subject matter experts. Risk factor weights were calculated and used in a geospatial analysis to calculate a risk value for each island nation (higher values indicate higher risk). Risk values ranged from 8.73 (Trinidad) to 1.57 (The Bahamas, Turks and Caicos Islands). All four countries that have documented occurrences of laboratory confirmed rabid bats were ranked highest (Trinidad and Tobago, Grenada, Cuba, Dominican Republic), as well as Haiti. The top five highest risk countries that currently have no reports of bat rabies include St. Vincent and the Grenadines, Jamaica, Puerto Rico, the Cayman Islands, and Dominica. This study reviews the inter-island movement potential of bats, designates areas of high risk for bat-associated rabies within the Caribbean islands, and demonstrates a need for further surveillance efforts in bat populations within islands that self-declare as rabies free.

## 1. Introduction

Evidence suggests rabies virus has persisted in the Americas for thousands of years, since before the pre-Columbian era [1]. Most of the 48 countries and territories in the Americas have significantly reduced the circulation of canine rabies variant within domestic dogs through dog vaccination campaigns, leash laws, and population management [2]. While canine rabies control efforts were very successful at reducing the number of dog-associated human rabies deaths and rabid dogs, there are limited tools available to control rabies in bat populations. In the Americas, reports of rabid bats have been stable in recent years [1,3,4]. Currently, all countries within the mainland of North, Central and South America are endemic for bat rabies virus variants [1,4,5,6,7]. As a result of the widespread control of canine rabies, Latin America now reports more human rabies cases transmitted by bats than cases transmitted by dogs [6,8]. Compared to other bat species in Latin America, *Desmodus rotundus* (the Common Vampire Bat), due to their hematophagous feeding habits, plays the most significant role in rabies outbreaks that affect humans and livestock [2,9]. However, rabies virus is enzootic throughout the Americas in many other bat species; 75 (22.5%) Latin American bat species have been confirmed as rabies positive, and only three species in the Americas are hematophagous [4]. In the Caribbean islands, hematophagous bats are only known to occur on the island of Trinidad [10]. However, numerous non-hematophagous bat species are found throughout the islands. Some of the non-hematophagous bat species found in the Caribbean islands are reservoir species of rabies on the Caribbean mainland (such as *Tadarida brasiliensis, Molossus molossus,* or *Artibeus jamaicensis*), and are also known to roost in human structures which can present a risk of rabies transmission to humans or domestic animals [4,11]. From the 28 island nations and dependent island territories in the Caribbean, four have documented rabies in bats, including Cuba, Dominican Republic, Grenada, and the island of Trinidad [4,12,13,14,15]. Several studies of bats in the Caribbean have demonstrated the ability to fly between islands. However, the degree and frequency of which various stochastic and deterministic factors affect these bat movements is not well described [16,17]. The paucity of bat movement or dispersal data presents epidemiologists, veterinarians, ecologists and public health workers with a challenge when attempting to understand and quantify the risk of bat rabies, and developing safe, effective, and common-sense policy for rabies prevention.

Recent reviews of research capacity and public health monitoring have identified several gaps in the knowledge regarding the incidence and prevalence of zoonotic infectious diseases in the region [10,18,19]. High-risk groups may be unaware of the potential risk of bat rabies in the Caribbean islands that self-declare as rabies free. A recent situational analysis of rabies status in the Caribbean revealed that 20 of 26 island nations reported themselves to be ‘rabies free’ or ‘non-endemic’ by World Health Organization criteria [10]. However, of these 20 island countries, only 20% indicate that a rabies surveillance and control program had been implemented [10]. As a result of the lack of surveillance data, limited information exists on actual bat rabies prevalence and distribution throughout the Caribbean region [10]. In one study conducted in Latin America [4], a significant positive association was found between the number of publications related to “bat rabies” and the number of bat species found to be rabies positive by country; highlighting the impact of surveillance effort in detecting rabies-positive bats. Furthermore, it is stated in a bat rabies serosurvey conducted in Grenada [14], that to the authors’ knowledge no studies (related to bat rabies surveillance) have been conducted in the Lesser Antillean islands which self-declare as rabies free.

Development of public health systems capable of detecting rabies virus in bats, should it be present, would require an extensive One Health effort. Recognizing that this effort is unlikely to be undertaken in the near future, a theoretical risk model of bat rabies presence in the Caribbean islands was created to provide a quantitative assessment to approximate the likelihood that rabies could be present yet unrecognized in Caribbean island nations. Theoretical risk models utilize the Analytic Hierarchy Process (AHP), along with geospatial modeling techniques that are guided by data obtained from literature reviews. The AHP [20] organizes judgments and logic in a structured approach to decision making and is the most commonly used utility-based method for multi-attribute decision making [21,22]. This analytical tool has been used to develop and evaluate surveillance systems and infectious disease risk with pathogens such as Zika virus, bovine spongiform encephalopathy, and other pathogens that threaten food safety [22,23,24]. Using the AHP and a review of bat movement potential, we aim to assess the likelihood that unrecognized bat-associated transmission of rabies virus (UBAT-RV) is occurring within the bat populations of the Caribbean islands. Given the absence of resources to implement large-scale rabies surveillance systems throughout the Caribbean, a theoretical risk model to evaluate UBAT-RV may additionally aid the development of surveillance programs.

## 2. Materials and Methods

### 2.1. Risk Factor Selection

The authors reviewed the available literature to assess potential risk factors that contribute to the presence of rabies in bat populations on an island ecosystem. Databases including PubMed, Journal Storage (JSTOR), and ScienceDirect were queried, including country and regional names within the Caribbean, and/or combinations of keywords including ‘island’, ‘bat’, ‘rabies’, ‘transmission’, ‘migration’, ‘flight’, ‘translocation’, ‘phylogeography’, ‘population genetics’, ‘metacommunity’, ‘homing’, ‘home range’, ‘foraging’, ‘disease ecology’, ‘surveillance’, ‘serosurvey’, ‘laboratory capacity’, ‘species richness’, ‘risk modeling’, ‘risk factors’, and ‘biogeography’. Species-specific keyword searches were also conducted. Caribbean rabies surveillance data was accessed from the Regional Information System for Epidemiological Surveillance of Rabies (SIRVERA), which is an online database that facilitates monthly reporting of rabies in the Americas, coordinated by the Pan American Health Organization [10]. Any potential risk factors that lacked available data for all Caribbean islands were not considered in the final model. After initial risk factor selection (described in the results section), five risk factors (RFs) were determined for use in this model: proximity to a country (mainland or island) with a documented occurrence of rabid bats (RF1), total number of bat species present on an island that have been documented as rabid on one or more of the Caribbean islands (RF2), total number of bat species present on the island (RF3), presence of rabies in carnivores (dogs and mongooses; RF4), and the area of the island (km^2^; RF5).

### 2.2. Questionnaire and Subject Matter Expert Selection

**Figure 1:**
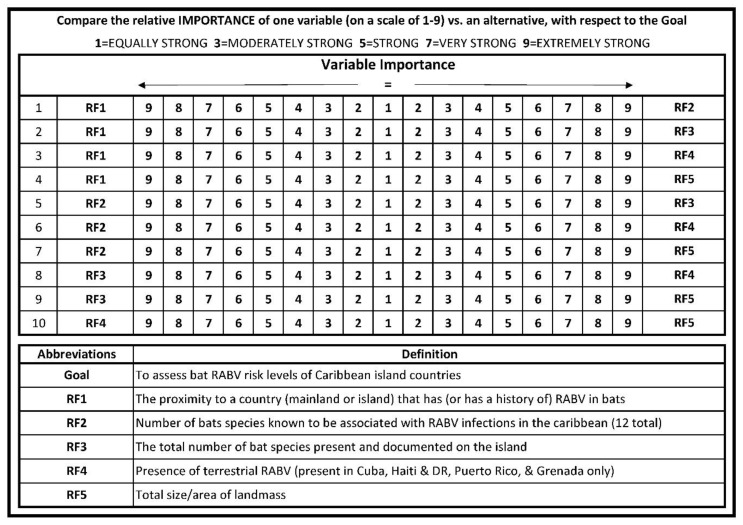
The pairwise comparison survey questionnaire sent out to subject matter experts to assess the risk factors that could potentially contribute to rabies presence in bat populations in the Caribbean islands.

A questionnaire consisting of pairwise comparison among the five risk factors was developed, based on the sample questionnaire proposed by Siddayao et al. 2014 [21]. The questionnaire (Figure 1) was then distributed to pre-selected subject matter experts (SMEs) via email. Inclusion criteria for SMEs included participants of a regional rabies working group, and or rabies public health expertise with research experience in bat-specific rabies ecology or epidemiology in the region. Credentials among the 8 solicited SMEs were as follows: 5 held doctor of veterinary medicine (DVM) degrees, 4 held doctor of philosophy (PhD) degrees with area of study ranging from biogeography to molecular genetics, 3 held master of public health (MPH) degrees (or equivalent), 4 were experts in North and or South American bat ecology and biology, 4 were experts in bat rabies epidemiology, 5 have served as rabies public health advisors, and 2 have served as heads of a World Organisation for Animal Health (OIE) reference laboratory. This questionnaire allowed SMEs to assign a number between 1 and 9 representing the relative importance of one risk factor relative to an alternative risk factor. This comparison was conducted for all possible pairs of risk factors. We then used the AHP to assemble matrices from each of the SMEs questionnaires. An aggregate matrix was assembled by averaging the value of each SME pairwise comparison to the nearest whole number, 1–9. We then calculated the sum of each matrix column within the aggregate matrix and normalized the matrix by dividing each cell value by the sum of that column. Finally, criteria weights for each risk factor were calculated by averaging the rows of the normalized matrix. The criteria weights of each risk factor calculated from the aggregate matrix were used in conjunction with the AHP plug-in tool [25] for ArcGIS software version 10.5 (ESRI, Redlands, California) to perform the geospatial analysis. To test for consistency between SME pairwise comparisons, the AHP plug-in tool also calculated a consistency ratio (CR).

### 2.3. Geospatial Analysis

Geospatial layers representing each of the five risk factors were generated. Each risk factor layer was created as a shapefile within the program ArcGIS and then rasterized and reclassified to perform the AHP analysis by assigning risk values (RVs; 1, 3, 5, 7, or 9) to each subset (or category) of risk factor data. To conduct the geospatial analyses, it was necessary to specify a grid-cell resolution of 1 km^2^. However, this 1 km^2^ resolution was not maintained in the final model outputs, as one RV will be assigned per country. The country-specific RV reported is the maximum value within the political boundary of an island nation. This is because rabies-free designation or the Centers for Disease Control and Prevention (CDC) traveler’s heath information is declared at the national level and not the level of individual islands. Rabid bats have not been reported from Haiti to date. However, it shares the island of Hispaniola with the Dominican Republic and is thus included in this analysis as a bat rabies-endemic country. Similarly, the island of Tobago also does not have any official reports of rabid bats and has no records of the presence of hematophagous bats [26,27]. However, Tobago was grouped with Trinidad, and analyzed at the national level in this study.

### 2.4. Risk Categorization

For the risk categorization of RF1, a shapefile was created in ArcGIS representing American mainland and Caribbean countries that have a documented presence of rabid bats [4], and then buffered to create five different distance ranges from each landmass. Each grid-cell within the area of the buffered landmasses had a value representative of its respective risk. The breakpoints between each distance range buffer (and their corresponding values) were determined after a review of bat flight distance ranges [28,29,30,31,32,33,34,35,36]. Broad distance ranges were set for each risk category due to limited information available concerning continuous flight travel distances of bat species in the Caribbean (required for over-water flight). However, continuous flight travel distances have been documented with the species *T. brasiliensis* (Brazilian Free-tailed Bat), which is present on most Caribbean islands [28]. This species was recorded in one study to have traveled 159.8 km in one continuous flight, and the average continuous flight distance of *T. brasiliensis* was 91 km [29]. In efforts to be conservative in the estimation of flight distance ranges, this average flight distance of 91 km was used as the approximate upper limit (51–100 km) for the median risk category (assigned a value of ‘5’). The longest continuous flight distance recorded for *T. brasiliensis* (159.8 km) was used to set the approximate range of the next highest distance range (101–200 km), grid-cells in this range were assigned a value of ‘3’. Any grid-cell greater than 200 km away from a landmass was assigned a value of ‘1’. Additional studies in bat flight capabilities from other species in the Caribbean and North America were also considered as proxies for the species in the Caribbean. One such study of the bat species *Myotis vivesi* (Fish-eating Bat) using Global Positioning System (GPS) tracking observed continuous average flight distances of 25.1 km [30]. Studies of *Leptonycteris yerbabuenae* (Lesser Long-nosed Bat) have demonstrated this bat flying distances of 53.5 km using GPS tracking [30], and 49.4 km using fluorescent powder tracking [31]. These flight distance measurements aided in determining the distance range for the second highest risk category (21–50 km), and grid-cells in this range were assigned a value of ‘7’. Flight speed of *Artibeus jamaicensis* (Jamaican Fruit-eating Bat), a species common across the Caribbean, has also been measured ranging from 15.4 km/h [32] to 24.1 km/h [33]. The flight speed measurements from these two studies (Mean = 19.75 km/h) provide an estimate for the flight capacity of this species and aided in the determining the distance range of the highest risk category (1–20 km). Grid cells within 1–20 km from a landmass were assigned a value of ‘9’. Actual distances flown by *A. jamaicensis* have only been recorded by few studies, one suggesting average distance traveled is 8 km per night [34].

For RF2, 12 bat species have been documented to be rabid on one or more of the Caribbean islands [4]. These bat species were considered as rabies-associated bat species (RABS), and include *Eptesicus fuscus* (Big Brown Bat)*, Eumops glaucinus* (Wagner’s Bonneted Bat)*, Tadarida brasiliensis* (Brazilian Free-tailed Bat)*, Diclidurus albus* (Northern Ghost Bat)*, Molossus molossus* (Pallas’s Mastiff Bat)*, Pteronotus davyi* (Davy’s Naked-backed Bat)*, Pteronotus parnellii* (Parnell’s Mustached Bat)*, Artibeus jamaicensis* (Jamaican Fruit-eating Bat)*, Artibeus lituratus* (Great Fruit-eating Bat)*, Carollia perspicillata* (Seba’s Short-tailed Bat)*, Desmodus rotundus* (Common Vampire Bat)*,* and *Diaemus youngi* (White-winged Vampire Bat). Only the latter two RABS are hematophagous bats—the remaining 10 are non-hematophagous bats. In the Caribbean, hematophagous bats are only known from the island of Trinidad, whereas the remainder of the Caribbean islands have no documented occurrences of hematophagous bats. Recent phylogenetic studies have proposed elevating some Caribbean subspecies of bat to species level based on genetic and morphological analysis [37,38,39]. However, to remain consistent with the current accepted taxonomy, recently proposed species will remain as subspecies, consistent with taxonomic assignments stated in Wilson and Reeder’s *Mammal Species of the World* [40]. A list of bat species per country was created using data from a study evaluating bat meta-community structure in the Caribbean islands [28]. We then created a shapefile of the Caribbean islands containing the total number of RABS present in each country. Breakpoints were determined using the Jenks natural breaks optimization method in the program R v.3.1.1 (http://www.R-project.org/). Islands containing more than 4 RABS were assigned a value of ‘9’ (only Grenada and Trinidad and Tobago), 3–4 RABS were assigned a value of ‘5’, and 1–2 RABS were assigned a value of ‘1’.

For RF3, we created a shapefile representative of the total number of bat species present within a country [28,41] and assigned values based on those totals. Breakpoints were determined using the Jenks natural breaks optimization method in the program R v.3.1.1 (http://www.R-project.org/). Each grid-cell containing more than 30 bat species (only the island of Trinidad) was assigned a value of ‘9’, 17–29 bat species were assigned a value of ‘7’, 11–16 species were assigned a value of ‘5’, 7–10 species were assigned a value of ‘3’, and less than 7 species were assigned a value of ‘1’.

For RF4, we identified countries that have non-bat rabies virus reservoir species (only canine rabies virus and mongoose rabies virus variant transmission cycles have been reported among Caribbean island nations) [10,13]. Each grid-cell containing rabies transmission in both dogs and the invasive small Indian mongoose (*Herpestes auropunctatus*) was assigned a value of ‘9’, transmission in either dogs or mongooses were assigned a value of ‘5’, and grid-cells with neither canine rabies virus nor mongoose rabies virus variants reported were assigned a value of ‘1’.

For RF5, we created a shapefile representative of the total area of each country’s landmass, using area data generated in ArcGIS from the shapefile of country boundaries sourced from ESRI, DeLorme Publishing Company, Inc. Breakpoints were determined using the Jenks natural breaks optimization method in the program R v.3.1.1 (http://www.R-project.org/). For this layer, countries that had an area of over 75,000 km^2^ were assigned a value of ‘9’, 25,000–75,000 km^2^ were assigned a value of ‘7’, 6000–25,000 km^2^ were assigned a value of ‘5’, 2500–6000 km^2^ were assigned a value of ‘3’, and any countries with an area of less than 2500 km^2^ were assigned a value of ‘1’.

### 2.5. Risk Value Calculation

**Figure 2:**
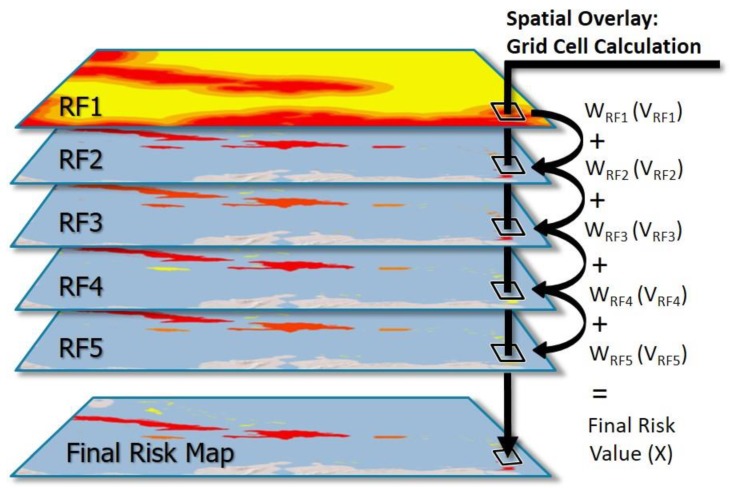
Risk map spatial overlay and grid cell calculation. Visualization of the spatial overlay of each risk factor (RF) raster file to create the final risk map. Within each grid cell of a RF layer, the grid cell value (V) was multiplied by the weight of its RF layer (W), and these values were then summed between all correlating grid cells to get a final risk value (X).

Following the risk categorization within all of the five RFs, we used ArcGIS to spatially overlay (Figure 2) each of the RF layers. This was accomplished using the raster calculator tool to calculate the sums of the value of each individual RF and spatially correlating grid cell (V), multiplied by the weight of each corresponding layer (W), to get a final value (X):

$$W_{1}\left(V_{1}\right)+W_{2}\left(V_{2}\right)+W_{3}\left(V_{3}\right)+W_{4}\left(V_{4}\right)+W_{5}\left(V_{5}\right)=X$$

The country-specific RV, and the symbology of the risk map reported in the final model outputs, depict the maximum value within the political boundary of each country.

### 2.6. Model Validation and High-Risk Threshold

To assess the accuracy of this risk model to reflect the likelihood of UBAT-RV, we excluded the effects of the Caribbean islands known to have documented occurrences of bat rabies (Trinidad and Tobago, Grenada, Cuba, and the island shared by Dominican Republic and Haiti), and developed a validation model. For the validation model, only proximity to the mainland (not including proximity to countries where bat rabies has been detected) was used for the RF1 raster. All other RFs were unchanged from previously described methods. If the RVs for the known islands where bat rabies has been detected remained high in the validation model, this would provide validity to the methods of this risk model. The lowest RV assigned to a country where bat rabies has been detected within this model was the threshold value for the designation of a high-risk country.

## 3. Results

Following the literature review and data search to identify RFs that could contribute to the unrecognized presence of rabies in an island population of bats, 10 potential RFs were identified by SMEs. Information associated with five of the potential RFs was either unknown for the Caribbean islands (i.e., species-specific bat movement capabilities) or there was insufficient data across all islands (i.e., surveillance effort); RFs that could not be applied to all Caribbean islands were excluded from model consideration. The five remaining RFs eligible for modeling were: proximity to a country (mainland or island) that has a documented occurrence of laboratory confirmed rabid bats (RF1), total number of bat species present in an island nation that has ever been documented as rabid, on one or more of the Caribbean islands (RF2), total number of bat species present on an island nation (RF3), presence of canine and/or mongoose rabies virus variants (RF4), and the land mass of the island (km^2^; RF5). In total, eight subject matter experts (SMEs) were solicited, and all responded to a standardized questionnaire (Figure 1). SME responses were used to create an aggregate pairwise comparison matrix, as shown in Table 1. The judgments of variable importance made by the SMEs were deemed to be consistent (CR < 0.10) within the aggregate pairwise comparison matrix. Variable weights were calculated for each RF and were ranked in the following order: RF2, RF1, RF3, RF4, and RF5. Risk factor rankings are displayed with their variable weight values in Table 2.

**Figure 3:**
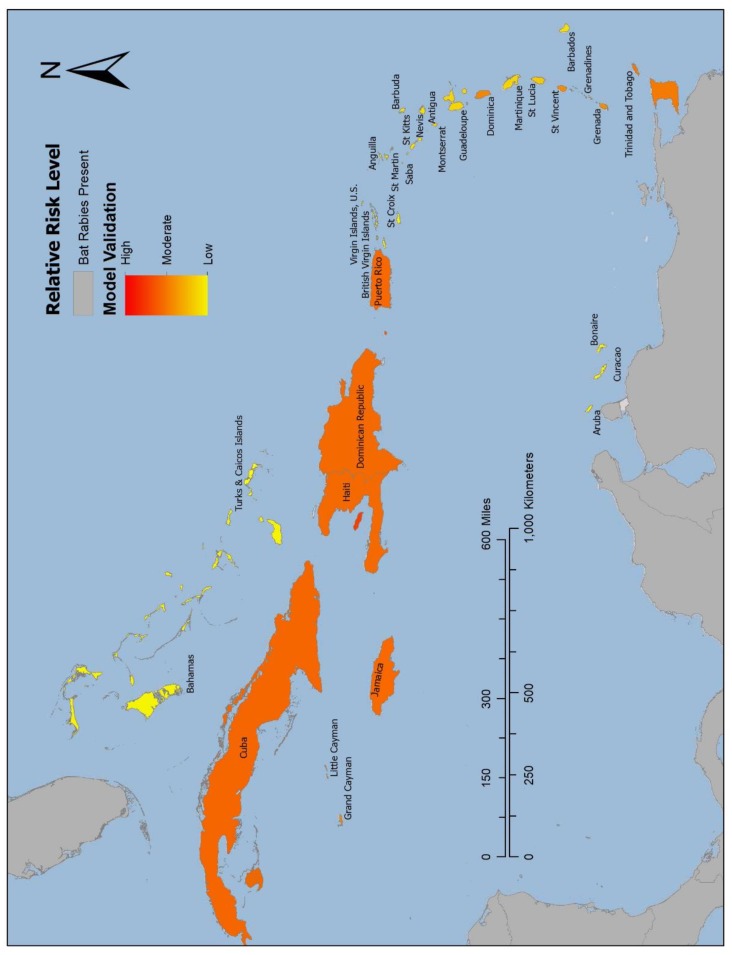
Map displaying results of the validation model. The validation model was created to assess the predictive capabilities of an Analytic Hierarchy Process (AHP) risk model to assign risk values to Caribbean islands representative of their likelihood of unrecognized bat-associated transmission of rabies virus (UBAT-RV). In the validation model, all RFs within the geospatial calculation signifying “having bat rabies” were removed from islands in which bat rabies has been detected. The validation model results determined that there was higher risk of enzootic rabies virus transmission in bats on islands that have documented laboratory confirmed rabid bats, signifying the predictive capabilities of an AHP risk model. Areas of high risk are symbolized by red and orange, and lower risk is symbolized yellow.

**Figure 4:**
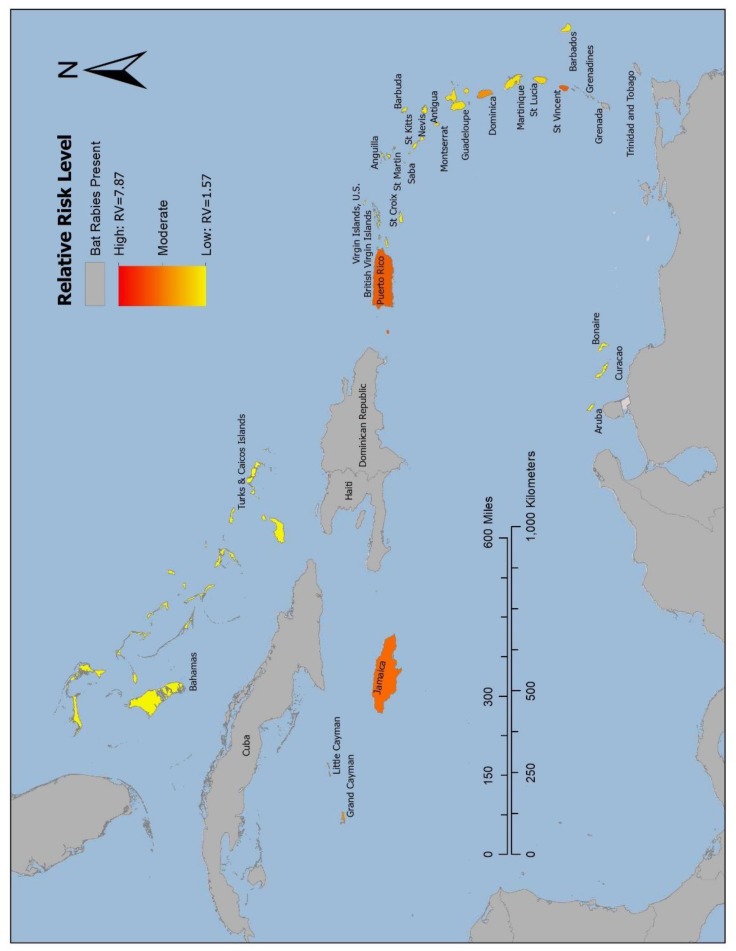
Risk map of bat rabies presence in the Caribbean islands. Choropleth map displaying relative risk level of bat rabies-free Caribbean island countries for presence of rabies virus in bat populations. Countries where bat rabies has been documented are shown in gray, high risk is symbolized by shades of red and orange, and lower risk is symbolized by shades of yellow.

The validation model (Figure 3) determined Cuba (6.17) and the island shared by the Dominican Republic and Haiti (6.08) as the highest risk, followed by Trinidad and Tobago (5.24), and Grenada (5.31). All these islands have documented occurrences of laboratory confirmed rabid bats [4,12,13,14,15]. Risk values attained from the validation model for each of the Caribbean countries where bat rabies has been detected are displayed in Table 3, along with the values from the risk model. The colorimetric scales in Figure 3 and Figure 4 are scaled to these values, reflective of the relative risk level.

The threshold for high risk was set by Grenada, as it was one of the countries where bat rabies has been detected that had the lowest RV in the validation model (RV = 5.31). The results of the risk model identified five of the self-declared bat rabies-free islands as high risk of UBAT-RV. High-risk island countries in order of highest RV are St. Vincent and the Grenadines (RV = 7.87), Jamaica (RV = 6.37), Puerto Rico (RV = 6.34), the Cayman Islands (RV = 5.92), and Dominica (RV = 5.35). Subsequent ranking and RV of Caribbean island countries are listed in Table 4. The choropleth map displaying the risk of UBAT-RV (Figure 4) is symbolized with the island countries in which bat rabies has been detected and mainland in gray, and the self-declared bat rabies-free countries range from shades of red and orange (high RVs) to shades of yellow (low RVs).

## 4. Discussion

### 4.1. Risk Factors and Weighting

The two highest weighted RFs in the AHP analysis were total number of rabies virus-associated bat species present in the country (RF2), followed by proximity to a country in which bat rabies has been detected (RF1), with 48% and 28% contribution to the model, respectively. SMEs valued these variables higher than the others in the pairwise comparison questionnaire. The total number of bat species (RF3) was weighted at 13% contribution to the model. It is important to note that the current bat species distributions represent estimations of presence, based from species occurrence records. Species distributions do not represent immutable barriers to bat dispersal, and are subject to change over time, which could alter the results of the risk model. This risk factor (RF3) is intended to be a proxy for the increased risk of rabies transmission between bats as a result of increasing the overall frequency of interspecific interactions. In some instances, rabies-infected bats have been observed as aggressors to other species [42], as well as exchanging rabies variants between migrating and non-migrating bat species [43].

The presence of canine rabies and/or mongoose rabies virus variants (RF4) and island land mass (RF5) were selected by SMEs to be the least important RFs for UBAT-RV, with 6% and 5% contribution to the model respectively. Regarding RF4, there was agreement among the SMEs that host-shift events from dogs or mongooses to bats would be very rare, and thus this RF contributes very little to the model. However, RF4’s limited contribution to the model may serve as a measure of undiagnosed bat-associated rabies cases in dogs and mongooses. The countries that have canine and mongoose rabies virus variants typically have less robust public health systems for conducting routine rabies virus characterization [10], and therefore it is possible that at least a small number of these rabies cases attributed to dog or mongoose rabies virus variants may be the result of infection from an unrecognized bat-associated rabies virus variant.

Regarding island land mass (RF5), this was selected after a review of island biogeography. According to MacArthur and Wilson’s equilibrium theory of island biogeography, larger islands should have higher immigration rates of bats, which may increase the potential for bat rabies dissemination between landmasses [44,45]. The relatively low weighting of this risk factor by the SMEs (5% contribution to the model) may be due in part to what is understood about gene flow barriers. Recent phylogenetic studies have demonstrated that there are monophyletic island bat populations in the Caribbean, as well as islands with significant gene flow (among other islands or the mainland) [37,38,46,47,48,49]. In some cases, there is no gene flow from small islands to neighboring large islands; in other cases, there is evidence for frequent movement between multiple small islands [37,38,46,47,48,49]. Therefore, island size alone may not have a large influence on bat movements and other barriers such as habitat or reproductive barriers may be more significant for certain bat species.

Proximity to a country in which bat rabies has been detected (RF1) was the second highest weighted risk factor contributing to the model, and the authors deemed it necessary to provide a brief overview of what is known about bat movements and movement potential. There are four species found on the majority of the Caribbean islands that are known to be capable of sustained flight over large bodies of water [28,46,48,49,50], namely, *Tadarida brasiliensis* (Brazilian Free-tailed Bat), *Artibeus jamaicensis* (Jamaican Fruit-eating Bat), *Molossus molossus* (Pallas’s Mastiff Bat), and *Noctilio leporinus* (Greater Bulldog Bat). Rabies virus has been confirmed in three of these strong flying species (excluding *N. leporinus*) in the Caribbean and elsewhere in the Americas [4], and it is worth noting that bats do not just move themselves but also their pathogens [16].

### 4.2. Evidence for Over-Water Bat Movements

Many natural and unnatural processes may play a role in the inter-island movements of bats, including natural migrations patterns to areas of higher resource availability (insect or fruit), predation of aerial insects along their migratory trajectories, formation of breeding aggregations, changing of roost site due to human disturbance, or seasonal tropical storms and hurricanes [35,50,51,52,53,54]. Translocation events caused by human activities have also been observed to occur such as bats roosting in or on shipping containers, ocean vessels, oil platforms, or aircraft [16,55]. Evidence for periodic over-water dispersal events in Caribbean bats include population genetics studies of *A. jamaicensis*, *Erophylla spp., T. brasiliensis, M. molossus, and Brachyphylla cavernarum* in the Lesser and Greater Antilles that suggest inter-island movement of these species does occur. Studies have also found occurrences of aberrant species in the Florida Keys originating from Cuba and the Bahamas [37,39,46,47,48,49,50,56]. Others have suggested that inter-island bat migration may be necessary to recolonize at least some of the Lesser Antillean islands and sustain bat populations after catastrophic events such as hurricanes [57,58,59].

Perhaps the strongest case for periodic over-water dispersal can be made for *T. brasiliensis*, an anthropophilic and gregarious species commonly known to transmit rabies in the Americas, and present on most Caribbean islands [28]. In one study conducted with a colony in Texas, this species was recorded to have traveled 159.8 km in a single flight without rest, over the course of 4.58 hours, reaching a top speed of 160.2 km/h [29]. This evidences *T. brasiliensis* as having the flight capacity to travel between any Caribbean island and its nearest neighbor, including travel to the most isolated island, Barbados, a distance of approximately 145 km from its nearest neighbor St. Lucia. Additionally, population genetics studies of *T. brasiliensis* in the Bahamas have shown population connectivity among island groups [49].

In Cuba, an insectivorous species of bat, *Eumops glaucinus* (Wagner’s Bonneted bat), was found to be infected with a rabies virus that is closely related to a clade of previously characterized rabies viruses circulating within hematophagous bats in Mexico [12]. Findings such as this support the concern that over-water movements of Caribbean bat species does indeed occur, and furthermore it is likely that these movements may occur periodically during the lifetime of certain bat species, representing a continuous potential for the spread of bat-associated rabies virus.

### 4.3. Bat Rabies Surveillance Efforts

The risk model developed herein has identified islands that have a high risk for UBAT-RV. However, these results do not refute nor confirm a rabies-free status within any Caribbean country. This risk model can be further assessed and validated through passive and/or active bat rabies surveillance and can inform the planning of future surveillance efforts. There are many studies that demonstrate that insular regions lacking rabies surveillance systems often remain unaware of the presence of this disease, particularly in wildlife. For example, Taiwan had been globally recognized as rabies-free since 1961. However, in 2011, the disease surveillance program was expanded to include native wildlife, and in 2013 three dead ferret badgers (*Melogale moschata*) tested positive for rabies virus by fluorescent antibody testing [60,61]. This new detection indicated that despite the lack of dog or human cases, rabies virus had silently remained in circulation within the wildlife population of Taiwan. In the Caribbean islands, where bat numbers are relatively low and some species are considered threatened or endangered, non-terminal serosurveys of bats could be enacted as a potential active surveillance method. Elsewhere, in a serosurvey conducted between 2005 and 2010 [62], researchers found serological evidence of widespread lyssaviruses in frugivorous and insectivorous bats on islands in the southwest Indian Ocean, including those self-declaring as rabies free. Additional examples of island countries discovering the presence of UBAT-RV or *Lyssavirus* only after active surveillance can be seen in the Philippines and Grenada [14,63,64].

### 4.4. Island-Specifc Risk

The results of this risk model highlight the island-specific potential for UBAT-RV in the Caribbean. Some countries, which self-identify as rabies free, were highlighted in this study as having a high or moderate relative risk of bat rabies being present, but unrecognized. The country with the highest RV from the model was St. Vincent and the Grenadines (RV = 7.87), likely due to the close proximity to countries in which bat rabies has been detected, Grenada and Trinidad and Tobago, and the relatively high number of bat species present (n = 13). The second and third highest risk countries identified by the model are Jamaica (RV = 6.37) and Puerto Rico (RV = 6.34) respectively. These are of particular concern, as they both are heavily populated, large islands that currently conduct limited active or passive rabies surveillance in bats. These are also the most densely human-populated islands, and studies elsewhere in the Caribbean have shown that urbanization level may be positively associated with rabies seroprevalence in bats [11]. The two lowest ranking island nations are the Bahamas (RV = 1.57) and Turks and Caicos Islands (RV = 1.57). These were likely listed as the lowest due to the comparatively low number of RABS on the islands, as well as total bat species [28]. Compared to other Caribbean islands, vertebrate species diversity in the Bahamas is very low due to its unique climatic and geologic history [65]. Although the Bahamas and Turks and Caicos Islands, as well as the remaining island nations listed in Table 4, were not identified as high risk in this risk model, adequate precautions should also be taken to prevent potential rabies exposures. It is important to note that Aruba (28 km north of Venezuela) is currently listed as lower risk in this model (RV=3.16), and the presence of *T. brasiliensis* or *A. jamaicensis* in Aruba is not documented in the available literature [41]. However, if either of these two species are subsequently reported as present in Aruba, the RV for this island would increase. 

Caribbean countries that were identified as high risk in this risk model are good candidates for the establishment of bat rabies surveillance systems. An increased understanding of the risk that comes from exposure to bats, treatment of bat exposures, and reporting of sick bats in these countries would serve as a benefit to public health programs. The CDC currently recommends that certain groups of U.S. travelers such as veterinarians, animal handlers, wildlife professionals, cavers and researchers should receive pre-exposure rabies immunization [66,67]. While the incidence of bat rabies may be low on some islands and sequestered in certain bat populations, the risk of rabies transmission to humans after contact with bats may still exist. Rabies is invariably fatal if exposures are not treated appropriately, therefore a high threshold of assurance must be met before international public health programs should acknowledge a self-declared rabies free status.

### 4.5. Study Limitations

Given the lack of quantitative data on bat rabies prevalence and distribution, the AHP offered a method by which we could assess the presence and risk of UBAT-RV in the Caribbean islands. This study was limited by the availability of accurate and reliable datasets across all countries in the Caribbean, which subsequently limited the number of risk factors that could be assessed in this study. A more robust methodology to bat rabies risk mapping can be performed if relevant data becomes available in the future, such as occurrence of human deaths with undiagnosed encephalitis, or if an increase in bat rabies surveillance capacity were developed. Additionally, further research could explore assessing gene flow and haplotype distribution among islands for use as a potential risk factor for bat rabies distribution. Studies that approximate the frequency of bat species introductions could help determine the frequency by which a bat-rabies free status needs to be re-assessed. This study relied on input from a panel of rabies public health professionals and rabies ecology experts, additionally experienced with bat ecology and bat rabies viruses. Outcomes of the risk model may vary based on responses of queried individuals with differing specializations. However, the high degree of agreement between the SME pairwise comparisons (CR < 0.10) provides additional validation of the method. 

The findings and conclusions in this report are those of the authors and do not necessarily represent the views of the Centers for Disease Control and Prevention, or the Pan American Health Organization/World Health Organization.

## Figures and Tables

**Table 1:** Aggregate pairwise comparisons matrix.

	Risk Factor Alternatives				
	RF1_a_	RF2_a_	RF3_a_	RF4_a_	RF5_a_
**RF1**	1	0.33	4	6	5
**RF2**	3	1	5	6	6
**RF3**	0.25	0.2	1	3	4
**RF4**	0.125	0.125	0.33	1	2
**RF5**	0.2	0.125	0.25	0.5	1

**Table 2:** Risk factors for bat rabies presence in the Caribbean islands, and variable weights.

Risk Factor		Variable Weight
**RF1**	Proximity to a country in which bat rabies has been detected	0.28
**RF2**	Total number of rabies virus-associated bat species	0.48
**RF3**	Total number of bat species present	0.13
**RF4**	Presence of rabies in carnivores	0.06
**RF5**	Island area (km^2^)	0.05

**Table 3:** Risk values from the risk model and validation model of islands with a documented occurrence of rabid bats.

Ranking	Island(s)	Model Risk Value	Validation Risk Value
1	Cuba	8.73	6.17
2	Dominican Republic	8.64	6.08
	Haiti	8.64	6.08
3	Trinidad and Tobago	8.25	5.69
4	Grenada	7.87	5.31

**Table 4:** Risk values of self-declared bat rabies-free islands from the risk model.

Ranking	Self-Declared Bat Rabies-Free Island(s)	Model Risk Value
1	St. Vincent and the Grenadines	7.87
2	Jamaica	6.37
3	Puerto Rico	6.34
4	Cayman Islands	5.92
5	Dominica	5.35
6	St. Lucia	3.75
7	Martinique	3.44
	Guadeloupe	3.44
	Montserrat	3.44
8	Antigua	3.18
	Barbados	3.18
	Anguilla	3.18
	Barbuda	3.18
	Nevis	3.18
	Saint-Martin	3.18
	Sint Maarten	3.18
	St. Kitts	3.18
	Saba	3.18
	Saint Barthelemy	3.18
	St. Eustatius	3.18
9	Aruba	3.16
10	British Virgin Islands	2.91
	U.S. Virgin Islands	2.91
11	Bonaire	2.40
	Curacao	2.40
12	The Bahamas	1.57
	Turks and Caicos Islands	1.57
